# Streptococcus pyogenes Transcriptome Changes in the Inflammatory Environment of Necrotizing Fasciitis

**DOI:** 10.1128/AEM.01428-19

**Published:** 2019-10-16

**Authors:** Yujiro Hirose, Masaya Yamaguchi, Daisuke Okuzaki, Daisuke Motooka, Hiroshi Hamamoto, Tomoki Hanada, Tomoko Sumitomo, Masanobu Nakata, Shigetada Kawabata

**Affiliations:** a Department of Oral and Molecular Microbiology, Osaka University Graduate School of Dentistry, Suita, Osaka, Japan; b Genome Information Research Center, Research Institute for Microbial Diseases, Osaka University, Suita, Osaka, Japan; c Institute of Medical Mycology, Teikyo University, Hachioji, Tokyo, Japan; University of Tartu

**Keywords:** *Streptococcus pyogenes*, necrotizing fasciitis, inflammatory environment, transcriptome, virulence factor, metabolism

## Abstract

Streptococcus pyogenes is a major cause of necrotizing fasciitis, a life-threatening subcutaneous soft-tissue infection. At the host infection site, the local environment and interactions between the host and bacteria have effects on bacterial gene expression profiles, while the gene expression pattern of S. pyogenes related to this disease remains unknown. In this study, we used a mouse model of necrotizing fasciitis and performed RNA-sequencing (RNA-seq) analysis of S. pyogenes M1T1 strain 5448 by isolating total RNA from infected hind limbs obtained at 24, 48, and 96 h postinfection. RNA-seq analysis results identified 483 bacterial genes whose expression was consistently altered in the infected hindlimbs compared to their expression under *in vitro* conditions. Genes showing consistent enrichment during infection included 306 encoding molecules involved in virulence, carbohydrate utilization, amino acid metabolism, trace-metal transport, and the vacuolar ATPase transport system. Surprisingly, drastic upregulation of 3 genes, encoding streptolysin S precursor (*sagA*), cysteine protease (*speB*), and secreted DNase (*spd*), was noted in the present mouse model (log_2_ fold change, >6.0, >9.4, and >7.1, respectively). Conversely, the number of consistently downregulated genes was 177, including those associated with the oxidative stress response and cell division. These results suggest that in necrotizing fasciitis, S. pyogenes shows an altered metabolism, decreased cell proliferation, and upregulation of expression of major toxins. Our findings are considered to provide critical information for developing novel treatment strategies and vaccines for necrotizing fasciitis.

**IMPORTANCE** Necrotizing fasciitis, a life-threatening subcutaneous soft-tissue infection, is principally caused by S. pyogenes. The inflammatory environment at the site of infection causes global gene expression changes for survival of the bacterium and pathogenesis. However, no known study regarding transcriptomic profiling of S. pyogenes in cases of necrotizing fasciitis has been presented. We identified 483 bacterial genes whose expression was consistently altered during infection. Our results showed that S. pyogenes infection induces drastic upregulation of the expression of virulence-associated genes and shifts metabolic pathway usage. In particular, high-level expression of toxins, such as cytolysins, proteases, and nucleases, was observed at infection sites. In addition, genes identified as consistently enriched included those related to metabolism of arginine and histidine as well as carbohydrate uptake and utilization. Conversely, genes associated with the oxidative stress response and cell division were consistently downregulated during infection. The present findings provide useful information for establishing novel treatment strategies.

## Abstract (precis)

Necrotizing fasciitis, a life-threatening subcutaneous soft-tissue infection, is principally caused by S. pyogenes. The inflammatory environment at the site of infection causes global gene expression changes for survival of the bacterium and pathogenesis. However, no known study regarding transcriptomic profiling of S. pyogenes in cases of necrotizing fasciitis has been presented. We identified 483 bacterial genes whose expression was consistently altered during infection. Our results showed that S. pyogenes infection induces drastic upregulation of the expression of virulence-associated genes and shifts metabolic pathway usage. In particular, high-level expression of toxins, such as cytolysins, proteases, and nucleases, was observed at infection sites. In addition, genes identified as consistently enriched included those related to metabolism of arginine and histidine as well as carbohydrate uptake and utilization. Conversely, genes associated with the oxidative stress response and cell division were consistently downregulated during infection. The present findings provide useful information for establishing novel treatment strategies.

## INTRODUCTION

Streptococcus pyogenes causes diverse diseases in humans, ranging from mild throat and skin infections to life-threatening invasive diseases, such as sepsis, necrotizing fasciitis, and streptococcal toxic shock syndrome. Streptococcal necrotizing fasciitis cases are clinically characterized by fulminant tissue destruction and rapid disease progression (1). Surgical treatment is required in the majority of affected patients, including amputations and intensive care. Although this infection has attracted increased research and clinical interest, the mortality rate remains high (2, 3). Investigation of the molecular pathogenesis of S. pyogenes related to necrotizing fasciitis is expected to lead to development of novel therapeutic strategies for effective treatment.

S. pyogenes typing historically has been conducted on the basis of M protein and T antigen (pilus major subunit) antigenicity. Sequence typing of the region encoding the hypervariable region of M protein has been widely applied and used to classify this organism into at least 240 *emm* sequence types (4–6) and 20 T serotypes (7, 8). In invasive cases reported in industrialized countries, S. pyogenes serotype M1 (*emm* 1) isolates are considerably more common than other serotypes (6, 9–11), with the M1T1 clone in particular being the most frequently isolated serotype from severe invasive human infections worldwide (12, 13).

At the infection site in the host, bacterial pathogens are exposed to drastically changing environmental conditions, such as host cells, tissues, and immune response, compared to laboratory growth conditions. However, no known previous study has compared *in vivo* and *in vitro* transcriptome findings of S. pyogenes.

Several previously reported transcriptome analyses have revealed the roles of S. pyogenes virulence-related regulators (14), including CovRS (15, 16) and CcpA (17–19). The transcriptome profile of S. pyogenes cells obtained from a mouse soft-tissue infection using microarray analysis indicated that S. pyogenes MGAS5005 (serotype M1) upregulated genes involved in oxidative stress protection and stress adaptation (20). Additionally, results of another microarray analysis of S. pyogenes MGAS5005 (serotype M1) demonstrated downregulation of glycolysis genes and induction of genes involved in amino acid catabolism as well as several types of virulence genes in human blood (21). Those results suggest that S. pyogenes changes its level of expression of virulence factors and metabolic pathways to adapt to the host environment.

Comprehensive understanding of bacterial transcriptomes *in vivo* will facilitate research aimed at developing therapeutic strategies or effective vaccine antigens. Although transposon-directed insertion site sequencing using a cynomolgus macaque model of necrotizing fasciitis was recently conducted (22), that method cannot be used to assess the expression level of genes. In addition, transcriptome analysis of S. pyogenes related to necrotizing fasciitis has not been performed to date. In the present study, we investigated transcriptome profiling of S. pyogenes M1T1 strain 5448 using a mouse model of necrotizing fasciitis from the acute to elimination phase, and we identified genes with expression consistently altered throughout the infection period. The novel information obtained thusly will be helpful to shed light on development of novel therapeutic strategies for this infectious disease.

## RESULTS

### Technique for high-yield purification of bacterial RNA from mouse tissue.

**FIG 1:**
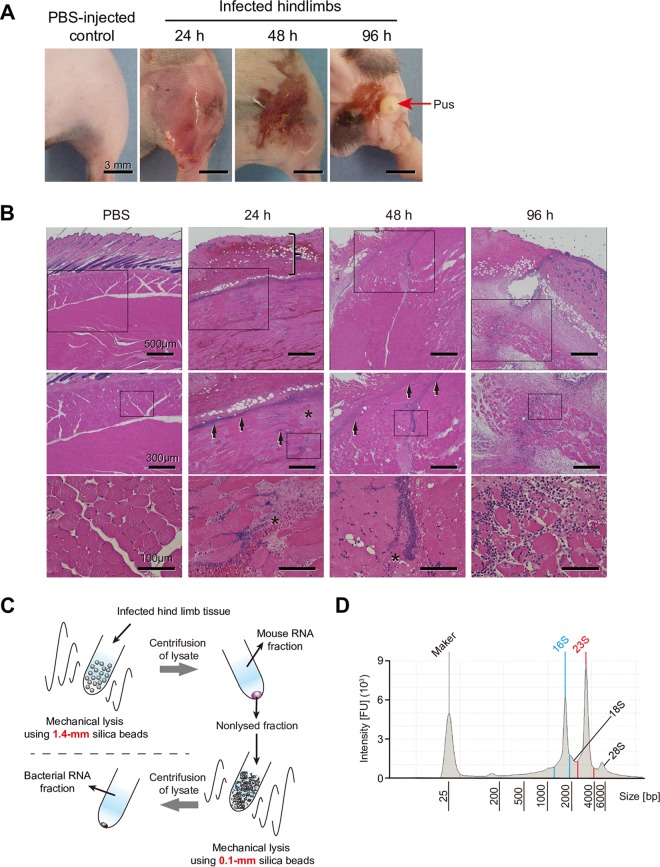
Features of mouse model of necrotizing fasciitis and workflow of bacterial RNA isolation. (A) Representative images of infected hindlimbs after inoculation with S. pyogenes M1T1 strain 5448. (B) Histopathological features of mouse model of necrotizing fasciitis. Hematoxylin and eosin staining of infected lesions at the indicated time points is shown, with higher-magnification images of the selected areas of the same sections also presented. At 24 h after infection, skin showed erosion of the epidermis and edematous thickening of the dermis (vertical bracket), as well as sparse inflammatory cell infiltration. At 24 and 48 h after infection, marked necrosis (asterisks) was observed, as the bacteria were primarily concentrated along the major fascial planes (arrows) in infected deep soft tissue. At 96 h after infection, sufficient inflammatory cell infiltration and elimination of pus (red arrows) from infected hindlimbs were observed. (C) Workflow of bacterial RNA isolation. Tissues were lysed with 1.4-mm silica spheres, and the mouse RNA fraction was removed after centrifugation. Pellets next were lysed with 0.1-mm silica spheres and centrifuged to obtain the bacterial RNA fraction. (D) Representative bioanalyzer profile of total RNA isolated from an infected hindlimb. 16S and 23S, bacterial rRNA peaks; 18S and 28S, mouse rRNA peaks. FU, fluorescence units.

We used a previously described mouse necrotizing fasciitis model, with minor modifications (23). At 24 and 48 h after infection, the mouse model was found to be histologically similar to human necrotizing fasciitis in terms of tissue necrosis, infection spread along fascial planes, inflammatory cell infiltration, hemorrhaging, and ulceration (24, 25) (Fig. 1A and B). Extensive scab formation was detected at 48 h, and elimination of pus from infected hindlimbs was observed at 96 h postinfection. At 96 h after infection, the weight of the mice tended to recover, and the bacterial burden at the infected site was also decreased (see Fig. S1 in the supplemental material). Therefore, we collected infected hindlimb samples at 24, 48, and 96 h after infection for further analyses. To obtain bacterial RNA from infected tissues, we established a suitable protocol using two types of beads (Fig. 1C), a method that allowed us to remove most mouse RNA from the samples and obtain high-yield purification of bacterial RNA (Fig. 1D).

### Similar gene expression patterns of S. pyogenes at three distinct time points in infected hindlimbs.

**FIG 2:**
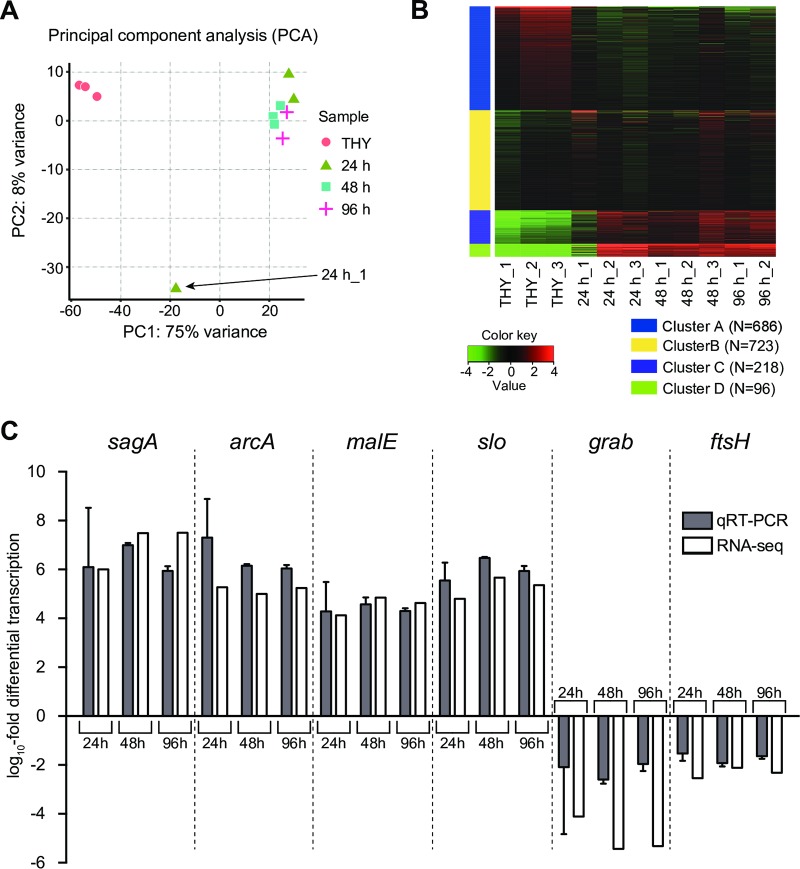
RNA-seq global reports. (A) Principal component analysis (PCA) plot of RPKM data from RNA-seq data set. (B) Heatmap of *k* means clustering of all genes (1,723 genes) in all samples (*k* = 4). The number of expressed genes in each cluster is indicated. The color key indicates Z score and displays the relative values of all tiles within all samples: green, lowest expression; black, intermediate expression; red, highest expression. Bacterial RNA-seq data at 24, 48, and 96 h postinfection were defined as the 24-h (24 h_1, 24 h_2, and 24 h_3), 48-h (48 h_1, 48 h_2, and 48 h_3), and 96-h (96 h_1 and 96 h_2) groups, respectively. Bacterial RNA-seq data of THY culture samples were defined as the control, termed the THY group (THY_1, THY_2, and THY_3). (C) Validation of RNA-seq data using qRT-PCR. The *x* axis shows selected genes subjected to qRT-PCR assays, and the *y* axis shows the log_10_ fold change relative to that of THY culture samples. qRT-PCR was performed with total RNA from the samples used for RNA-seq. Data from two or three independent qRT-PCR assays, each performed in triplicate, were pooled and normalized. *rpoB* was used as the internal control. Vertical lines represent the means ± standard errors (SE).

We performed RNA-sequencing (RNA-seq) analysis of S. pyogenes cells isolated from infected hindlimbs at 24, 48, and 96 h postinfection. RNA-seq data of S. pyogenes during the exponential growth phase in THY medium (Todd-Hewitt broth plus yeast extract) were defined as the control. To assess the global gene expression profiles of the samples, we performed principal component analysis (PCA) (Fig. 2A), hierarchical clustering analysis (Fig. S2), and *k* means clustering analysis (Fig. 2B) using the RNA-seq data. PCA and hierarchical clustering analysis results showed that bacterial RNA expression patterns in THY culture samples formed clusters and samples from infected tissues that were well separated. The 24 h_1 sample had a global gene expression profile that was distant from the profiles of the other samples, whereas the heatmap of *k* means clustering showed that the gene expression profile of 24 h_1 was at least partially similar to that of samples from infected tissues, as indicated in clusters A and B. Furthermore, *k* means clustering findings suggested that most samples from infected hindlimbs demonstrated changes in global mRNA transcript patterns in opposite directions compared to the THY group. Results of quantitative real-time PCR (qRT-PCR), performed using primers shown in Table 1, validated the RNA-seq data (Fig. 2C). Six genes that altered the expression during infection were chosen for validation. Four upregulated genes encoding streptolysin S precursor (*sagA*), arginine deiminase (*arcA*), maltodextrin-binding protein (*malE*), and streptolysin O (*slo*) and two downregulated genes encoding macroglobulin-binding protein (*grab*) and a cell division protein (*ftsH*) were evaluated.

**TABLE 1:**
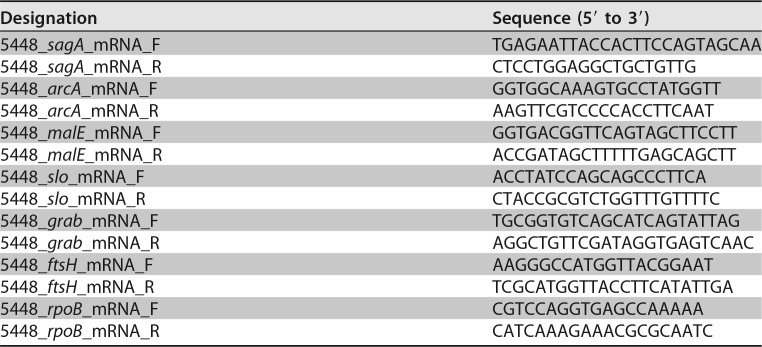
Quantitative real-time PCR primers used in this study

### Consistently altered bacterial genes at three time points in mouse necrotizing fasciitis model.

**FIG 3:**
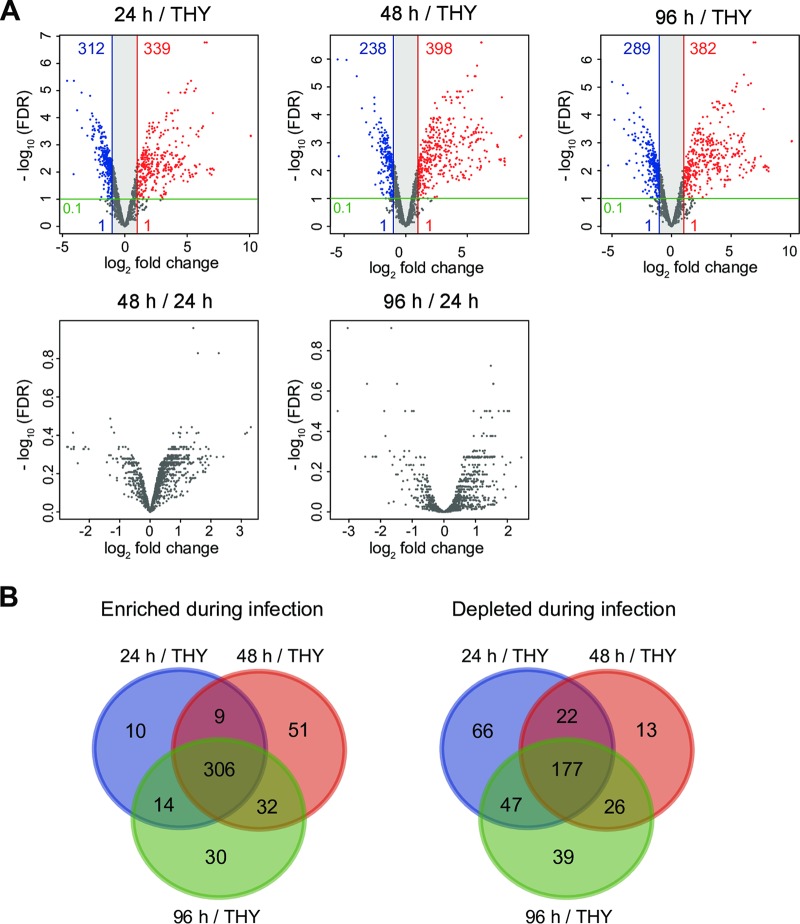
Differentially expressed genes showing consistent alteration in hindlimbs at each infection phase. (A) Volcano plots show gene expression differences under the comparison conditions indicated in each figure. Colored circles indicate significantly upregulated (red) and downregulated (blue) genes (absolute log_2_ fold change, >1; adjusted *P* < 0.1). (B) Three-way Venn diagram illustrating bacterial genes consistently altered during infection relative to the THY condition (24 h versus THY, 48 h versus THY, and 96 h versus THY). The findings showed that 306 transcripts were consistently enriched *in vivo* (log_2_ fold change, >1; adjusted *P* < 0.1), and 177 transcripts were consistently downregulated *in vivo* (log_2_ fold change, <−1; adjusted *P* < 0.1).

Differentially expressed genes (DEGs; absolute log_2_ fold change of >1, adjusted *P* value of <0.1) were detected in comparisons between S. pyogenes cells obtained from infected tissues and those grown in THY broth (Fig. 3A). Data set S1 shows DEG details as well as the following information for all genes: gene identifier, gene name, gene-associated function, log_2_ fold change, adjusted *P* value, and reads per kilobase per million mapped reads (RPKM) value. In comparisons of the 48-h and 24-h groups and the 96-h and 24-h groups, no DEGs were detected (Fig. 3A), while only 4 DEGs were detected in comparisons between the 96-h and 48-h groups (Data set S1). These results indicate that S. pyogenes expressed similar genes at the three examined time points in the present mouse model of necrotizing fasciitis. To identify genes consistently enriched or downregulated in the necrotizing fasciitis model, we produced Venn diagrams using DEGs from comparisons of the 24-h and THY groups, 48-h and THY groups, and 96-h and THY groups (Fig. 3B), which identified 483 (28.0%) of all 1,723 genes as consistently altered bacterial genes in infected hindlimbs (Data set S2). Among those 483 altered genes, 306 were upregulated and 177 downregulated at all three time points. As the results shown in Fig. 2A suggest that 24 h_1 is an outlier, we also reanalyzed our data without 24 h_1 data (Fig. S3A and Data sets S3 and S4) and compared them with the original results (Fig. S3B and Data set S5). However, major differences that affected our interpretation of the original results were not identified.

### Marked upregulation of genes encoding virulence factors.

**FIG 4:**
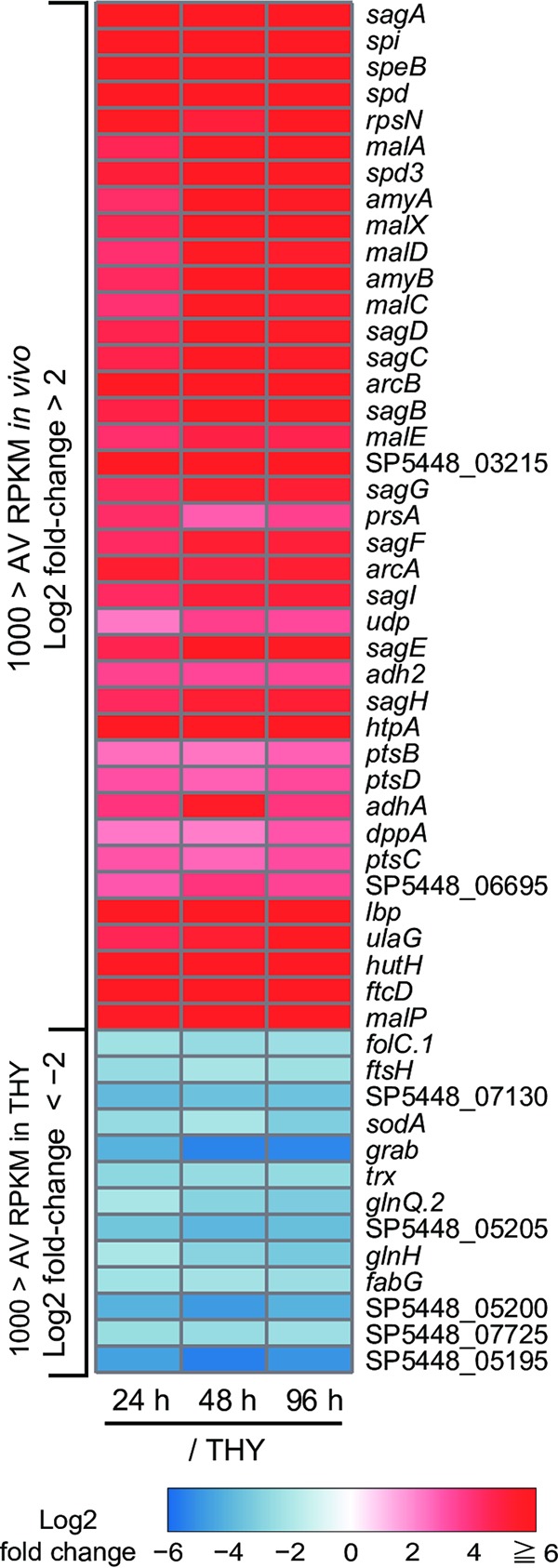
Heatmap of significantly altered genes *in vivo*. Heatmap of consistently and significantly enriched (log_2_ fold change, >2; average RPKM *in vivo*, >1,000) and downregulated (log_2_ fold change, <−2; average RPKM in THY, >1,000) genes. Color scale indicates enrichment (red) and depletion (blue) during infection. Values represent the log_2_ fold change between indicated conditions, and genes are arranged in descending order of expression level (average of RPKM values *in vivo*). AV RPKM, average RPKM.

Genes shown to be consistently enriched featured a high proportion of those encoding virulence factors, such cytolysins (*sagA-I* and *slo*), nucleases (*spd*, *spd3*, and *sdaD2*), and cysteine protease (*speB*), as well as factors involved in immune evasion (*endoS*, *spyCEP*, *scpA*, and *sic*), superantigens (*speA* and *smeZ*), and adhesins (*fbaA*, *lbp*, and *emm*) (Table 2 and Data set S2). Surprisingly, the RPKM values for the gene encoding streptolysin S precursor (*sagA*), *speB*, the SpeB inhibitor-encoding gene (*spi*), and *spd* were extremely high and consistently ranked within the top four (Fig. 4 and Data set S1). Compared with their expression under the THY condition, *sagA*, *speB*, *spi*, and *spd* were expressed in mouse necrotizing fasciitis at log_2_ fold changes of >6.0, >9.3, >9.4, and >7.1, respectively. In contrast, the gene encoding macroglobulin-binding protein (*grab*) was markedly downregulated at all three time points. Finally, the hyaluronic acid synthesis operon (*hasABC*) was significantly upregulated in the 48-h and 96-h groups, whereas no significant difference was detected in the 24-h group.

**TABLE 2:** Expression levels of selected genes/operons/regulon

Gene category and SP5448 number	Gene name^*a*^	Encoded function(s)	Log_2_ fold change^*b*^		
			24 h	48 h	96 h
Virulence factors					
870	*nga*	NAD glycohydrolase	5.05*	5.79*	5.49*
880	*slo*	Pore-forming cytotoxin	4.80*	5.67*	5.35*
1845	*endoS*	Immunoglobulin-modifying protein	3.91*	5.10*	4.66*
2470	*sda1*	Streptococcal nuclease D	4.08*	5.30*	4.93*
3755	*spd3*	Streptococcal extracellular nuclease 3	5.11*	6.27*	5.70*
4075	*grab*	Macroglobulin-binding protein	−4.11*	−5.43*	−5.32*
1702	*speA*	Superantigen	1.11*	1.80*	2.23*
6700–6740	*sagABCDEFGHI*	Secreted cytotoxin	4.69*	5.76*	5.62*
7800	*spyCEP*	IL-8-degrading protease	4.12*	5.37*	5.71*
8645	*smeZ*	Superantigen	1.24*	1.20*	1.25*
8690	*lbp*	Laminin-binding surface protein	6.56*	6.13*	7.04*
8710–8730	*fbaA*, *scpA*, *sic*, *emm*	Mga virulence regulon	2.00*	2.94*	3.54*
8800	*speB*	Streptococcal cysteine protease	10.09*	9.38*	10.11*
8820	*spd*	Streptococcal nuclease B	7.06*	7.80*	7.74*
9365–9375	*hasABC*	Hyaluronic acid capsule biosynthesis	0.79	1.08*	1.39*
Carbohydrate utilization					
1210–1240	*nanH*	Sialic acid production and catabolism	3.84*	3.84*	4.08*
1830–1855	*pmi*, *scrK*, *endoS*, *scrAB*	Sucrose transport and catabolism	1.94*	3.12	2.23
2540–2570	*lacABD*	Galactose transport and catabolism	3.53*	3.27*	3.83*
4190–4210		Cellobiose transport	2.64*	3.47*	3.54*
4275–4305	*malACDX*, *amyAB*	Cyclodextrin transport and catabolism	4.29*	6.23*	5.74*
5660–5675	*ptsABCD*	Mannose/fructose/sorbose transport	2.67*	2.37*	2.84*
8320–8350	*lacABDEFG*, *lacC.2*	Lactose transport and catabolism	2.95*	2.39*	3.81*
Amino acid utilization					
3210–3235	*arcABCD*	Arginine catabolism	5.88*	5.67*	5.84*
8955–8995	*hutDGHIU*, *ftcD*, *fchA*, *fhs.2*	Histidine catabolism	6.38*	7.22*	7.27*
Peptide transport					
1400–1420	*oppABCDF*	Oligopeptide transporter	0.69	0.88*	0.62
8655–8675	*dppABCDF*	Dipeptide permease	1.79*	1.93*	2.78*
Oxidative stress response					
3280	*dpr*	DNA protection during starvation protein	−0.86	−0.44	−1.19
3875	*sodA*	Superoxide dismutase	−2.58*	−2.07*	−3.12*
7040	*gpoA*	Glutathione peroxidase	−1.40*	−1.32*	−1.19*
8945	*ahpC*	Alkyl hydroperoxide reductase	−0.64	−0.33	0.31
Trace metal transport					
560–570	*adcRCB*	Zinc transport	1.86*	1.87*	2.29*
1895–1920	*shr, shp, siaABCD*	Iron and manganese transport	3.73*	4.11*	4.29*
2520–2530	*copZAY*	Copper transport	1.18*	0.23	1.93*
7665–7675	*mtsABC*	Iron and manganese transport	0.56	0.72	0.18
7885–7900	*fhuGBDA*	Iron and manganese transport	1.97*	2.06*	1.64*
8685–8690	*htpA*, *lbp*	Zinc transport	6.48*	6.14*	6.97*
Sodium and proton transport					
805–840	*ntpABCDEFKI*	V1-V0 (V)-ATPase transport system	3.11*	4.49*	4.67*
6640–6675	*atpABCDEFGH*	F1-F0 (F)-ATPase transport system	−0.97	−1.14*	−1.38*

a Only gene names annotated by PATRIC are shown.

b The expression values of 24-h, 48-h, and 96-h groups relative to those of the THY group are shown. Log_2_ fold changes of operons or regulons are shown as mean log_2_ fold changes in transcript levels for all genes. An asterisk indicates significant difference: *, *P* < 0.1. In operons or regulons, asterisks indicate that a significant difference was confirmed for all included genes.

### Upregulation of carbohydrate uptake and utilization genes.

**FIG 5:**
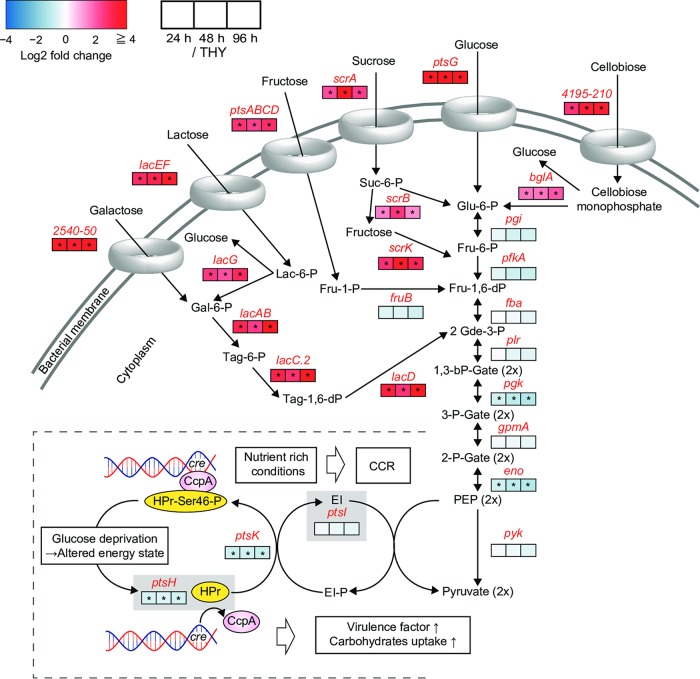
Central carbon metabolism and catabolite control protein CcpA. The pathway shown was constructed for S. pyogenes MGAS5005 based on the BioCyc database. Metabolite names are written in black and gene names in red. Log_2_ fold changes of the 24-h (left), 48-h (center), and 96-h (right) groups with respect to the THY group are indicated in the color scale boxes. Color scale indicates enrichment (red) and depletion (blue) during infection. An asterisk indicates significant difference: *, *P* < 0.1. In operons or regulons, asterisks indicate that a significant difference was confirmed for all included genes. Log_2_ fold change values of operons or regulons shown are mean log_2_ fold changes in transcript levels of all genes. The phosphocarrier protein HPr (*ptsH*) is phosphorylated at Ser46 by the kinase HPrK (*ptsK*) through the cytoplasmic enzyme EI (*ptsI*), which allows HPr-Ser46-P to dimerize with the carbon catabolite protein CcpA and elicit carbon catabolite repression by binding to catabolite response elements in promoter sequences (26).

Consistently enriched genes also included most of those encoding ATP-binding cassette (ABC) transporters or phosphoenolpyruvate-phosphotransferase system (PTS) molecules responsible for carbohydrate transport (Fig. 5, Table 2, and Data set S2). In the glycolysis pathway, the expression of *pgk* (encoding phosphoglycerate kinase) and *eno* (encoding enolase) showed a slight decrease, while the RPKM values of these genes consistently remained at >1,500. Despite sufficient expression of glycolysis system molecules in the infected hindlimbs, the carbohydrate transport systems exhibited an overall increase. Shelburne et al. reported that the carbon catabolite protein CcpA upregulates the expression of most operons encoding transporters of carbohydrates, such as glucose, lactose, maltodextrin, mannose, fructose, cellobiose, lactose, galactose, and sialic acid, under glucose-limiting conditions (18). Moreover, our results indicated that genes encoding phosphocarrier protein (*ptsH*) and its kinase (*ptsK*) were consistently downregulated (Fig. 5). When Gram-positive bacteria are exposed to glucose, the phosphocarrier protein (HPr) is phosphorylated at Ser46 by its kinase, HprK, which allows phosphorylated HPr to dimerize with CcpA, and then the dimerized proteins bind to catabolite response elements present in promoter sequences and elicit carbon catabolite repression (26). These findings raise the possibility that S. pyogenes was relieved from carbon catabolite repression in the present mouse model of necrotizing fasciitis.

Mutations of the CovRS virulence regulator derepress several different kinds of virulence genes, leading to greater mortality in mouse models of invasive infection (27, 28). S. pyogenes M1T1 strain 5448, used in our study, possesses an intact *covRS* locus. Therefore, we investigated whether the mouse-passaged S. pyogenes cells in this study gained a CovS mutation, as previously reported (29). As a result, only 0.2% to 0.4% of animal-passaged S. pyogenes possessed a CovS sequence mutation (Fig. S4 and S5). Thus, the low percentage of CovRS mutants might not have had a major impact on the present results.

### Drastically enhanced arginine and histidine metabolism in infected hindlimbs.

**FIG 6:**
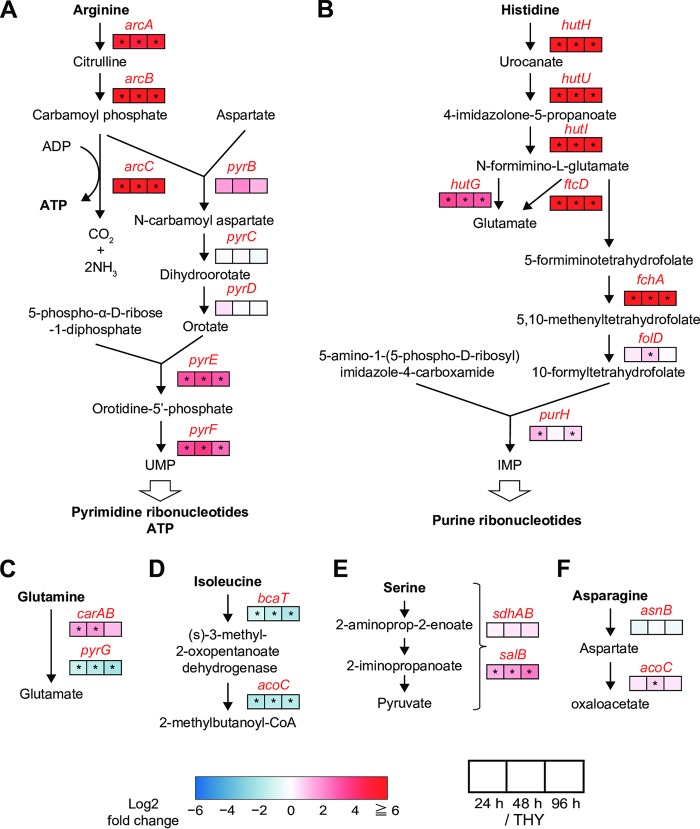
Significant enhancement of arginine and histidine metabolic pathways. (A) Arginine deiminase and pyrimidine nucleotide *de novo* synthesis pathways. (B) Histidine degradation and purine nucleotide *de novo* biosynthesis pathways. (C to F) Glutamine (C), isoleucine (D), serine (E), and asparagine (F) degradation pathways. Pathways for S. pyogenes MGAS5005 were constructed based on the BioCyc database. Metabolite names are written in black and gene names in red. Log_2_ fold changes of the 24-h (left), 48-h (center), and 96-h (right) groups with respect to the THY group are indicated in color scale boxes. Color scale indicates enrichment (red) and depletion (blue) during infection. An asterisk indicates significant difference: *, *P* < 0.1.

S. pyogenes has been shown to be auxotrophic for at least 15 amino acids (30). Consistently enriched genes identified in the present study included operons for metabolism of arginine (*arcABCD*), histidine (*hutDGHIU*, *ftcD*, *fchA*, and *fhs.2*), and serine (*salB*) (Table 2, Fig. 4 and 6, and Data set S2). Conversely, bacterial genes encoding proteins for isoleucine metabolism (*bcaT* and *acoC*) were consistently downregulated in the infected hindlimbs (Fig. 6). The operon for the dipeptide transporter *dppABCDF*, involved in uptake of essential amino acids (31), was also upregulated in the infected hindlimbs (Table 2), while the expression of *dppA*, which encodes a dipeptide-binding protein, was remarkably enhanced (log_2_ fold change of >2.25) (Fig. 4 and Data set S2).

The mean fold changes in transcript levels (i.e., mean log_2_ fold change values) for all genes in the operons for arginine and histidine metabolism were >5.67 and >6.38, respectively. In S. pyogenes, the arginine deiminase pathway (*arcABCD*) has been reported to supplement energy production, help protect against acid stress, and compete with arginine-dependent NO production by host cells in the subcutaneous layer (32). Another critical role of arginine metabolism is to serve as the source of UMP (Fig. 6A), whereas histidine metabolism is connected to the synthesis of IMP (Fig. 6B). These functions cooperate with pyrimidine and purine metabolism for the synthesis of DNA and RNA. The consistently enriched genes also included some related to pyrimidine and purine metabolism, such as those encoding pyrimidine biosynthesis (*pyrE* and *pyrF*), ribonucleotide reductase (*nrdE.1*, *nrdF*, and *nrdD*), uridine phosphorylase (*udp*), and DNA polymerase III epsilon subunit (*dnaQ*) (Data set S2). These results suggest that bacterial synthesis of nucleic acids was active in the infected hindlimbs, although we also observed repression of certain genes related to cell division, such as those encoding cell division proteins (*ftsA*, *ftsZ*, and *ftsH*), amino acid ligases (*murD* and *murG*), phospho-*N*-acetylmuramoyl-pentapeptide transferase (*mraY*), and RNase III (*rnc*) (Fig. 4 and Data set S2).

### Host-induced bacterial stress responses.

Genes encoding superoxide dismutase (*sodA*) and glutathione peroxidase (*gpoA*) were consistently downregulated in the infected hindlimbs (Table 2 and Data set S2). SodA and GpoA act to neutralize endogenous and exogenous peroxides, which has been shown to contribute to detoxification of reactive oxygen species *in vitro* (33, 34). Our results suggest that the S. pyogenes cells in the infected hindlimbs were not exposed to substantial oxidative stress compared to stress encountered during aerobic growth.

Transition metals are involved in several crucial biological processes necessary for pathogens to survive, proliferate, and cause disease in their environmental niche. In S. pyogenes, contributions to virulence are made by the homeostasis of metals, including iron, manganese (35), and zinc (36), whereas host tissues exploit this phenomenon and combat invading pathogens by restricting the availability of essential metals by using transferrin (iron), lactoferrin (iron), and calprotectin (manganese and zinc) (37). We found that S. pyogenes upregulated genes involved in iron and manganese transport (*shr*, *shp*, *siaABCD*, and *fhuGBDA*) and zinc transport (*adcRCB*, *htpA*, and *lbp*) in the present mouse model of necrotizing fasciitis (Table 2 and Data set S2).

### Altered expression of virulence-related transcriptional regulator genes.

The expression of most virulence genes in S. pyogenes is under the control of two-component signal transduction systems (TCSs) and transcriptional activators/repressors (14). Although phosphorylation is recognized as a key modification by which regulators exert regional transcriptional control (26, 38, 39), alternation of regulator gene expression levels could also influence the degree of regulation.

**FIG 7:**
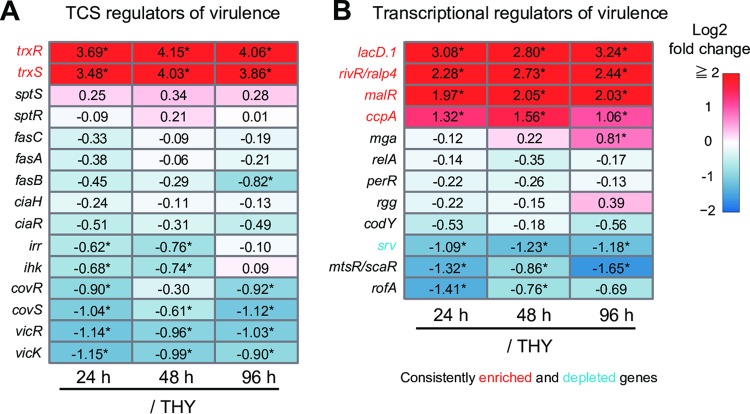
Expression levels of genes encoding virulence-related transcriptional regulators. (A and B) Two-component signal transduction systems (TCSs) (A) and transcriptional regulators of virulence (B). Log_2_ fold changes of the 24-h (left), 48-h (center), and 96-h (right) groups with respect to the THY group are indicated in color scale boxes. Numbers in the frame represent measured log_2_ fold change values. Color scale indicates enrichment (red) and depletion (blue) during infection. An asterisk indicates significant difference: *, *P* < 0.1.

The present findings showed that S. pyogenes altered the expression of several genes encoding virulence-related regulators (Fig. 7). Consistently enriched genes included the TCS *trxSR* operon as well as genes encoding carbohydrate-sensitive regulators (*lacD.1* and *ccpA*), a member of the RofA-like protein type family of stand-alone virulence-related regulators (*rivR*, also known as *ralp4*), and a maltose repressor (*malR*). Conversely, the only consistently downregulated regulator gene was that encoding the streptococcal regulator of virulence (*srv*), although other regulators also tended to show downregulation, including genes for CovRS (*covRS*), the metabolic control regulator VicRK (*vicRK*), a metalloregulator (*mtsR*, also known as *scaR*), and the RofA regulator (*rofA*).

## DISCUSSION

We report here comprehensive gene expression analyses of S. pyogenes in a mouse model of necrotizing fasciitis. For RNA-seq analysis of bacteria in host tissues, deep sequencing has been previously used to obtain a sufficient number of reads (40). However, the present protocol is simple and inexpensive and appears to effectively enable *in vivo* RNA-seq analysis of Gram-positive bacteria without deep sequencing (see Table S1 in the supplemental material). Furthermore, we also analyzed the transcriptome profiles of S. pyogenes at three distinct time points during infection. Our results indicated that S. pyogenes drastically and consistently upregulated the expression of virulence-associated genes and also shifted metabolic pathway usage in the present mouse model of necrotizing fasciitis. In particular, high levels of expression of *sagA*, *speB*, and *spd* were detected during infection. In contrast, S. pyogenes in infected hindlimbs downregulated genes associated with oxidative stress response and cell division compared to bacterial cells in THY cultures at the mid-logarithmic phase.

RNA-seq analysis revealed that *sagA*, *spi*, *speB*, and *spd* were extremely upregulated in the mouse model of necrotizing fasciitis compared to levels in bacterial culture medium. Streptolysin S (SLS; encoded by *sagA-I*) and SpeB (encoded by *speB*) are widely recognized virulence factors of S. pyogenes (41). SLS is involved in cellular injury, phagocytic resistance, and virulence in murine subcutaneous infection models (42, 43). Both SLS and SpeB also promote S. pyogenes translocation via a paracellular route by degrading epithelial junctions (44, 45). SpeB is a secreted cysteine protease that functions to degrade a wide variety of host proteins, including complement components and cytokines, and has functions to assist S. pyogenes for escape from host immune response (46–50). Moreover, SpeB has been shown to substantially contribute to bacterial virulence in necrotizing myositis mouse models (23, 51). The *spi* and *speB* genes are cotranscribed (52). The *spi* gene encodes a specific SpeB inhibitor, Spi, to protect bacterial cells from the activity of residual unsecreted SpeB. The present findings demonstrated that DNases encoded by *sda1*, *spd3*, and *spd* were also markedly upregulated. Sda1 allows S. pyogenes to escape killing in neutrophil extracellular traps and contributes to the virulence of murine subcutaneous infection (53, 54). The level of expression of *spd*, which encodes streptodornase B or mitogenic factor 1, was ranked fourth of all genes examined in the present study, while a previous report also noted its contribution to the virulence of S. pyogenes (serotype M89) (55). Although S. pyogenes possesses various virulence factors (12, 41, 56), these four genes showed extremely high levels of upregulation in our infection model. These findings should be helpful for revealing therapeutic targets for necrotizing facilities.

We also detected a drastic upregulation of virulence genes encoding histidine triad protein (HtpA) (57) and laminin-binding protein (Lbp) (58), which may also provide valuable insight regarding the utility of these molecules as therapeutic targets. Although a mouse intraperitoneal infection model was used, we found that HtpA functions as an effective vaccine antigen against S. pyogenes (59). Furthermore, a previous analysis of sera from patients with uncomplicated S. pyogenes infection or rheumatic fever indicated detectable humoral response against recombinant S. pyogenes Lbp (60).

The 483 genes shown to be consistently altered in the present study overlap 150 low-glucose-induced genes reported for strain HSC5 (serotype 14) (17), including upregulation of genes encoding molecules involved in carbohydrate uptake and metabolism, arginine metabolism, V-type ATP synthase, and lactate oxidase and downregulation of genes encoding molecules related to oxidative stress response and cell division. As for expression of genes encoding virulence factors, we observed an overlap of upregulation of genes for SLS, streptolysin O, and Spd and downregulation of the GRAB gene. These findings suggest that S. pyogenes in the infected hindlimbs encountered a glucose-poor environment and relieved carbon catabolite repression (26).

Mutations in *covRS* of S. pyogenes serotype M1 (strain 5448) have been reported to enhance virulence during subcutaneous infection in mice and might be responsible for loss of SpeB expression (54). Graham et al. also reported that serotype M1 S. pyogenes (MGAS5005) showed reduced levels of the *speB* transcript during growth in human blood (21). However, in the present study, the gene encoding SpeB was drastically upregulated in the mouse model of necrotizing fasciitis (log_2_ fold change of >9.38). The environment encountered by S. pyogenes in necrotizing fasciitis is considered to be distinct from that in blood or subcutaneous tissue. Although blood acidity is maintained in a narrow range of approximately pH 7.4 in living organisms, inflammatory loci are typically associated with an acidic environment (61). Moreover, our results suggested that S. pyogenes encounters glucose deprivation in necrotizing fasciitis. In S. pyogenes, *speB* expression in the early stationary phase can be substantially suppressed by glucose and buffered pH (62). Generally, the stationary phase of bacterial growth is evidenced by glucose depletion and medium acidification. Thus, an environment similar to the bacterial stationary phase might have induced the strong expression of *speB* seen in the present results.

Graham et al. characterized the MGAS5005 (serotype M1) transcript profile in a mouse soft-tissue infection model (subcutaneous infection) using wild-type and Δ*covR* strains (20). Interestingly, relative to the wild-type strain, the Δ*covR* strain exhibited drastic upregulation of *sagA* (18-fold), *speB* (2,053-fold), and *spd* (6-fold) in the model, and normalized expression levels of these 3 genes in the Δ*covR* strain were ranked eighth, second, and fifth, respectively. In the present study, S. pyogenes in the mouse model of necrotizing fasciitis also showed extremely high normalized expression levels of *sagA* (ranked first), *speB* (third), and *spd* (fourth) among 1,723 genes. One of the classic signs of acute inflammation is heat, and muscle temperature is considered to be higher than skin temperature (63). Although Graham et al. showed that subcutaneous infection causes necrosis of subcutaneous tissue and fascia in some areas of muscle, the mouse model of necrotizing fasciitis used in our study demonstrated extensive myositis. S. pyogenes appears to encounter higher temperatures during myositis than during subcutaneous infection, which might lead to its distinct transcriptome profiles.

Arginine, histidine, and serine are present at high concentrations (approximately 1,000, 500, and 1,000 μM, respectively) in human muscle tissue, while the concentration of isoleucine is low (approximately 150 μM) (64). Under a glucose-poor environment, S. pyogenes may use amino acids present at high concentrations in muscle tissue. Since a supply of amino acids is essential for protein and nucleic acid synthesis, the arginine and histidine metabolic pathways are likely to be enhanced, as observed in the present findings, for pathogenicity to be exerted in necrotizing fasciitis. Moreover, for the uptake of essential amino acids, the operon encoding the dipeptide transporter (DppABCDF) was found to be consistently upregulated in the infected hindlimbs. Deletion of S. pyogenes *dppA* results in a reduction of *speB* expression to one-eighth of its original level (serotype M49, strain CS101) (31). Thus, *dppA* upregulation might contribute to the drastically increased expression of *speB*.

Recently, Zhu et al. identified genes required for a cynomolgus macaque model of necrotizing fasciitis using transposon-directed insertion site sequencing (22). Serotype M1 (MAGS2221) genes necessary for infection identified in that study overlap certain genes found to be upregulated in our study, such as those for carbohydrate metabolism (*glgP* and *malM*), arginine metabolism (*arcABCD*), and putative or known transporters (valine, *braB*; zinc, *adcBC*; SLS, *sagGHI*). RNA-seq analysis and transposon-directed insertion site sequencing have distinct advantages. RNA-seq analysis allows for evaluation of relative expression levels among all examined genes. However, transcript levels do not always accurately predict the importance of a particular gene for a phenotype, while transposon-directed insertion site sequencing can directly identify genes contributing to the fitness of S. pyogenes in infected tissues. Therefore, assessments using results obtained with both methods are considered to be more effective for consideration of therapeutic targets.

No previous study has reported transcriptome profiling of S. pyogenes in necrotizing fasciitis. The present findings revealed that S. pyogenes cells in our mouse model of necrotizing fasciitis exhibited substantially altered global transcription compared to those cultured under *in vitro* conditions. S. pyogenes might attempt to acquire nutrients from destroyed tissues by markedly upregulating the expression of such toxins as SLS, SpeB, and Spd. Furthermore, genes encoding molecules involved in carbohydrate and amino acid utilization, as well as metal transporter genes, were shown to be upregulated in the infected mouse hindlimbs. We also consider that the present protocol for isolating bacterial RNA from infected tissues at high concentrations will facilitate investigations that utilize global gene expression analyses of bacteria in an *in vivo* host environment. Future studies may be conducted to explore new therapies based on bacterial kinetics *in vivo* by exploiting the present data or use of our methods. Accumulation of *in vivo* gene expression profiles will provide useful information necessary for establishing novel treatment strategies and identification of effective vaccine antigens.

## MATERIALS AND METHODS

### Bacterial strains and culture conditions.

S. pyogenes M1T1 strain 5448 (accession no. CP008776) was isolated from a patient with toxic shock syndrome and necrotizing fasciitis and considered to be a genetically representative globally disseminated clone associated with the invasive infections (65). S. pyogenes strain 5448 was cultured in a screw-cap glass tube (Pyrex; Iwaki Glass, Tokyo, Japan) filled with Todd-Hewitt broth (BD Biosciences, San Jose, CA) supplemented with 0.2% yeast extract (THY) (BD Biosciences) at 37°C in an ambient atmosphere. For growth measurements, overnight cultures of S. pyogenes strain 5448 were back diluted 1:50 into fresh THY and grown at 37°C, with growth monitored by measuring optical density at 600 nm (OD_600_).

### Necrotizing fasciitis examinations.

We used 10-week-old male C57BL/6J mice (Charles River Japan, Inc., Kanagawa, Japan) for the necrotizing fasciitis experiments, as previously described (23). After culturing S. pyogenes until the mid-exponential phase (OD_600_ of ∼0.5), THY was replaced with phosphate-buffered saline (PBS) and the bacterial suspensions were stored in a refrigerator (–80°C). Viable cell counts of the suspensions were determined by plating diluted samples on THY blood agar. Mice were shaved and hair was removed through chemical depilation (Veet; Oxy Reckit Benckiser, Chartes, France), and then the mice were inoculated intramuscularly on both sides of the hindlimbs with 2 × 10^7^ CFU suspended in 100 μl of PBS, which was prepared immediately before infection by diluting frozen stocks. Mice injected with PBS served as a noninfected control.

Mice were euthanized at 24 (*n* = 3), 48 (*n* = 3), or 96 (*n* = 2) h after infection by a lethal intraperitoneal injection of sodium pentobarbital, and then infected hindlimbs were collected. The left hindlimbs were immediately placed in RNAlater (Qiagen, Valencia, CA) and stored at –80°C until use for RNA isolation, whereas the right hindlimbs were fixed with formalin, embedded in paraffin and sectioned, and stained with hematoxylin and eosin as previously described (66).

### RNA isolation.

Thawed tissues were placed in lysing Matrix D microtubes containing 1.4-mm silica spheres (Qbiogene, Carlsbad, CA) with RLT lysis buffer (RNeasy fibrous tissue minikit; Qiagen, Hilden, Germany) and homogenized at 6,500 rpm for 45 s using a MagNA lyser (Roche, Mannheim, Germany). The lysate was centrifuged, and the obtained pellet was resuspended in lysing Matrix B microtubes containing 0.1-mm silica spheres (Qbiogene) with the RLT lysis buffer and homogenized at 6,500 rpm for 60 s using the MagNA lyser. The final lysate was centrifuged and bacterial RNA was isolated from the collected supernatant with an RNeasy fibrous tissue minikit, according to the manufacturer’s guidelines, and stored at –80°C (Fig. 1C).

### RNA-seq and data analysis.

RNA integrity was assessed using a 2100 Bioanalyzer (Agilent Technologies, Santa Clara, CA) (Fig. 1D). For RNA-seq, 5 μg of bacterial RNA was treated for rRNA removal using a Ribo-Zero rRNA removal kit (mouse and bacteria) (Illumina Inc., San Diego, CA). Directional RNA-seq libraries were created using a TruSeq RNA Sample Prep kit, v2 (Illumina Inc.), according to the manufacturer’s recommendations. Libraries were sequenced using Illumina NovaSeq 6000 and HiSeq 2500 systems, with 100-bp paired-end reads obtained (Macrogen, Daejeon, South Korea). Data were generated in the standard Sanger FastQ format, and phred-type quality scores of Q30 were used for quality trimming. RNA-seq reads were mapped against the S. pyogenes strain 5448 genome (accession number CP008776) using the commercially available CLC Genomics workbench, v. 9.5.2 (CLC Bio, Aarhus, Denmark). Differential expression and global analyses of RNA-seq expression data were performed using iDEP (http://ge-lab.org/idep/) (67), with the RPKM value of each sample determined. Results were visualized using volcano plots (iDEP) and Venn diagrams (http://bioinformatics.psb.ugent.be/webtools/Venn/). EdgeR log transformation was used for clustering and PCA (iDEP). Hierarchical clustering was visualized using the average linkage method with correlation distance (iDEP). Data were also clustered by use of *k* means with 1,723 genes (*k* = 4) (iDEP). We classified the DEGs into functional categories based on the bacterial bioinformatics database and analysis resource PATRIC (www.patricbrc.org) (68), which is integrated with information from VFDB (http://www.mgc.ac.cn/VFs/) (69), Victors (70), the subsystems technology toolkit (*RASTtk*) (71, 72), and the KEGG map (73). Genes were also classified into pathways based on the BioCyc database (74). Transcriptomic (RNA-seq) data are summarized in Data set S1.

### qRT-PCR assay.

Total bacterial RNA was isolated from infected hindlimbs, and then cDNA was synthesized using a Superscript VILO cDNA synthesis kit (Thermo Fisher Scientific, Waltham, MA, USA). Real-time RT-PCR analysis was performed using a StepOnePlus real-time PCR system (Applied Biosystems, Foster City, CA, USA) and Toyobo SYBR green RT-PCR master mix kit (Toyobo Life Science, Osaka, Japan). Data for *rpoB* were used as the internal control. Primers are listed in Table 1.

### Data availability.

Raw reads determined in this work were deposited into the DDBJ sequence read archive (DRA) under accession number DRA008246.

## Supplementary Material

Supplemental file 1

Supplemental file 2

Supplemental file 3

Supplemental file 4

Supplemental file 5

Supplemental file 6
