# Supplementary material for: Streptococcus pyogenes Transcriptome Changes in the Inflammatory Environment of Necrotizing Fasciitis
Source: Appl Environ Microbiol. 2019 Oct 16;85(21):e01428-19. doi: 10.1128/AEM.01428-19 (PMC6803311; doi:10.1128/AEM.01428-19)
Supplement: Supplemental file 1 [file AEM.01428-19-s0001.pdf]

Supplementary Information

Streptococcus pyogenes transcriptome changes in inflammatory environment of necrotizing fasciitis

Yujiro Hirose[1*], Masaya Yamaguchi[1], Daisuke Okuzaki[2], Daisuke Motooka[2], Hiroshi Hamamoto[3], Tomoki Hanada[1], Tomoko Sumitomo[1], Masanobu Nakata[1], Shigetada Kawabata[1*]

[1]Department of Oral and Molecular Microbiology, Osaka University Graduate School of Dentistry, Suita, Osaka 5650871, Japan

[2]Genome Information Research Center, Research Institute for Microbial Diseases, Osaka University, Suita, Osaka 5650871, Japan

[3]Institute of Medical Mycology, Teikyo University, Hachioji, Tokyo 1920352, Japan

*** Correspondence to:**

Yujiro Hirose: yujirohirose@dent.osaka-u.ac.jp

Shigetada Kawabata: kawabata@dent.osaka-u.ac.jp

Table of contents

1. Materials and Methods 2
2. Supplementary Table 3
   S1: Information regarding read counts of each sample 3
3. Supplementary Figures 3
   S1: Mice recovered from necrotizing fasciitis at 96 h after infection 3
   S2: Heat map showing clustering of all genes 4
   S3: Re-analyzed results after exclusion of 24 h_1 and comparisons with original results...... 5
   S4: SpeB activity of animal-passaged *S. pyogenes*. 6
   S5: Amino acid sequence alignment of CovS of clones containing *covS* mutation........ 7
4. Supplementary References 8

1. Materials and Methods

Determination of bacterial burden in infected tissues

Using a mouse model of necrotizing fasciitis, mice were euthanized at 24 (n = 3), 48 (n = 3), and 96 (n = 2) h after infection using a lethal intraperitoneal injection of sodium pentobarbital, then infected hindlimbs were collected. Samples were homogenized using a MagNA Lyser Instrument (Roche Applied Science, Penzberg, Germany) for 30 s at 5000 rpm. Bacterial burden in tissues was determined after plating serial dilutions of tissue homogenates, with corrections for differences in tissue weight. *S. pyogenes* organisms were routinely grown in 5% sheep blood THY (Todd-Hewitt broth plus 0.2% yeast extract) agar plates and cultured at 37°C. To isolate SpeB-negative colonies, serial diluted samples were also plated in THY milk/agar (Todd-Hewitt broth plus 1% yeast extract and 3% skim milk).

covS gene sequencing of SpeB-negative clones

S. pyogenes hypervirulent strains with an inactivated CovR/S system have been reported to lack detectable SpeB (1). *S. pyogenes* M1T1 strain 5448, used in the present study, contains an intact *covRS* locus. Therefore, we investigated whether mouse-passaged bacteria in this study retains retained the CovS system mutation, as previously noted (2). Briefly, SpeB-negative clones were defined by lack of a visual lytic (clearing) zone in THY milk/agar after 48 h of incubation at 37°C (Figure S4). Genomic DNA of SpeB-negative clones was obtained. Primers specific for the *covS* region (forward - GTCTATATTCGTTATCTCCGCGG, reverse -GCATCAGCTTCTAACCAGTTGT) were used to amplify the gene product by PCR. To identify mutations, nucleotide sequences were obtained using the same primers.

2. Supplementary Table

Table S1: Information regarding read counts of each sample

Sample name	Library name in DRA008246	Accession	Total number of read counts	Number of reads mapped against *S. pyogenes* (accession: CP008776)	Percentage of mapped reads
THY_1	THY_log_biological replicate 1	DRX165268	64,469,066	59,306,942	91.99
THY_2	THY_log_biological replicate 2	DRX165269	40,177,570	35,936,214	89.44
THY_3	THY_log_biological replicate 3	DRX165270	59,496,522	54,873,650	92.23
IF24_1	24 h_infec_biological replicate 1	DRX165271	57,352,832	26,803,194	46.73
IF24_2	24 h_infec_biological replicate 2	DRX165272	50,591,344	10,682,784	21.12
IF24_3	24 h_infec_biological replicate 3	DRX165273	56,335,776	25,590,488	45.42
IF48_1	48 h_infec_biological replicate 1	DRX165274	58,136,032	14,042,848	24.16
IF48_2	48 h_infec_biological replicate 2	DRX165275	58,613,638	7,852,860	13.40
IF48_3	48 h_infec_biological replicate 3	DRX165276	58,982,882	10,048,430	17.04
IF96_1	96 h_infec_biological replicate 1	DRX165277	61,977,716	1,679,132	2.71
IF96_2	96 h_infec_biological replicate 2	DRX165278	58,754,526	1,692,290	2.88

3. Supplementary Figures

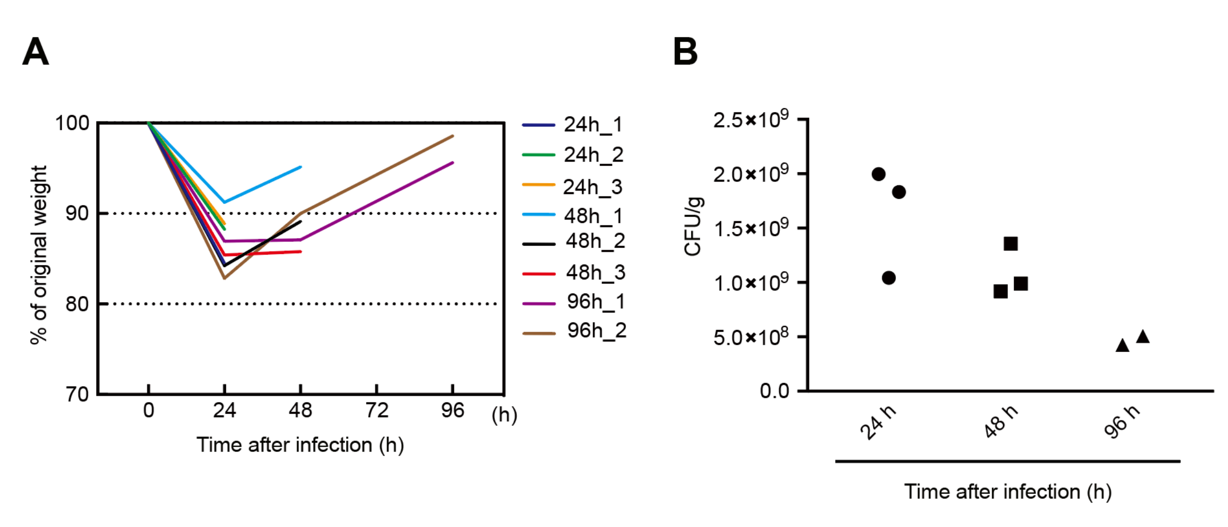

Figure S1. Mice recovered from necrotizing fasciitis at 96 h after infection. Ten-week-old male C57BL/6J mice were infected with 2×10^7 CFU of *S. pyogenes* intramuscularly in the hindlimb. (A) Body weight was monitored until sample collection. Body weight at 0 h was considered to be 100%. (B) CFU of *S. pyogenes* in infected hindlimb samples.

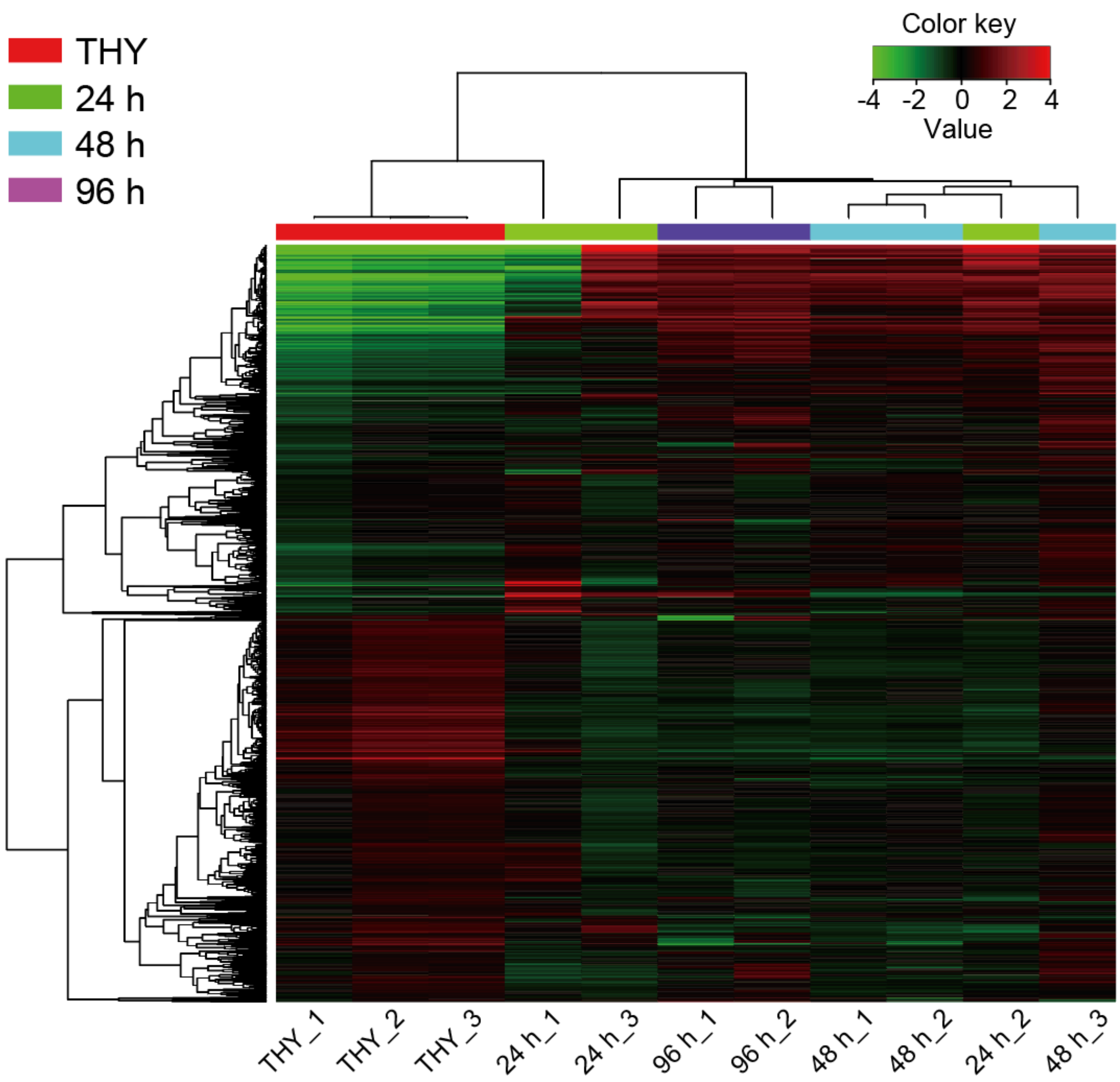

Figure S2. Heat map showing clustering of all genes (1723 genes). Each column represents a sample and each row a gene. Clustering was performed using iDEP (http://ge-lab.org/idep/) with edgeR log transformation of reads per kilobase million (RPKM). Hierarchical clustering was illustrated using the average linkage method with correlation distance. Color-coding is based on edgeR log-transformed RPKM values. Color key indicates Z-scores, which are displayed as relative values of all tiles within all samples. Green indicates the lowest level, black an intermediate level, and red the highest level of expression.

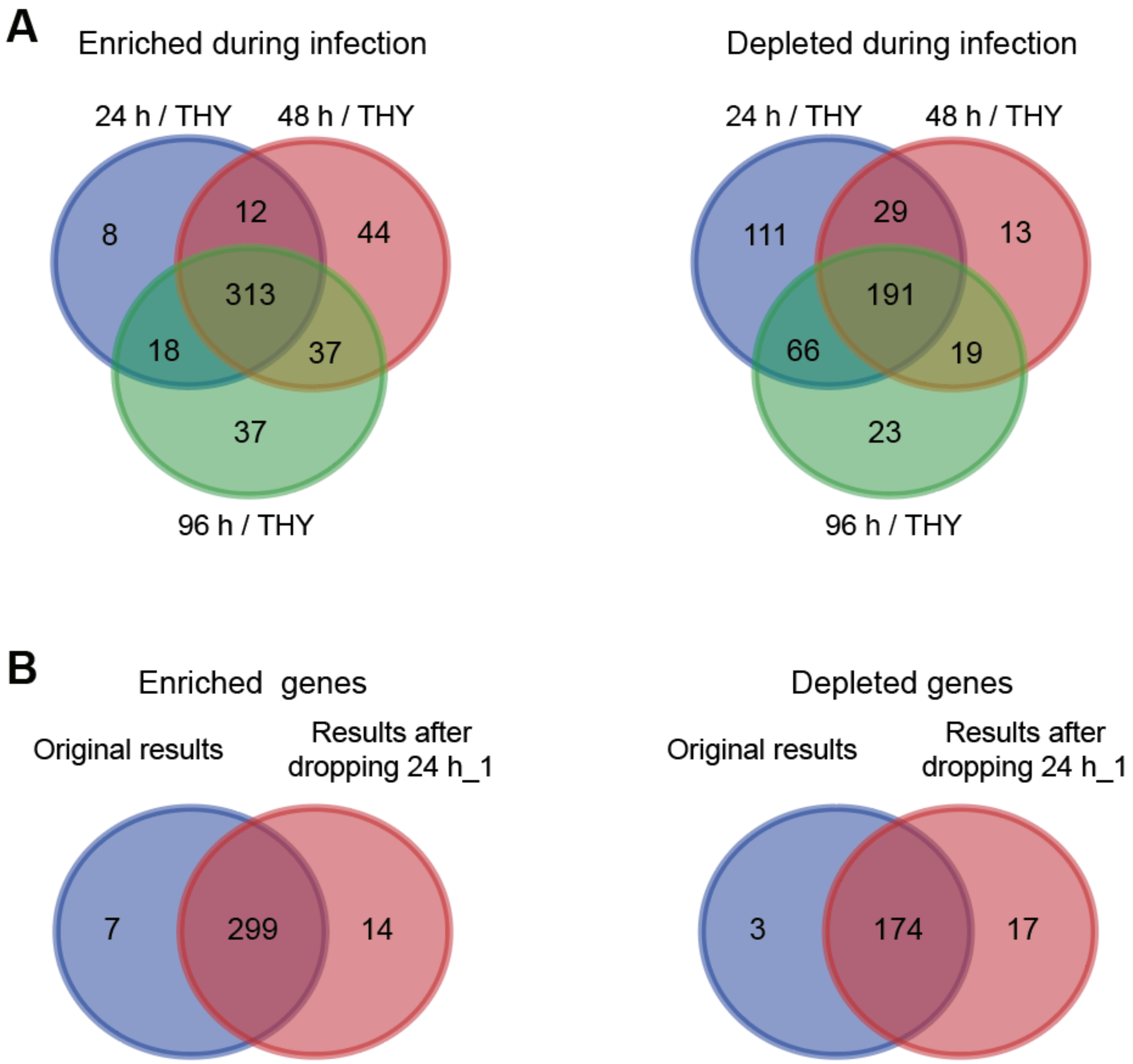

Figure S3. Re-analyzed results after exclusion of 24 h_1 and comparison with original results. (A) Three-way Venn diagram illustrating genes consistently altered during infection relative to the THY condition (24 h vs. THY, 48 h vs. THY, 96 h vs. THY). We found that 313 transcripts were consistently enriched *in vivo* (log_2 fold-change >1; adjusted P <0.1) and 191 transcripts were consistently downregulated *in vivo* (log_2 fold-change <-1; adjusted P <0.1). (B) Genes overlapping between original results and results after dropping 24 h_1.

A

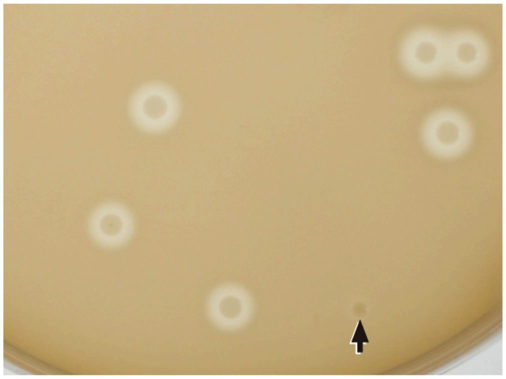

B

Frequency of SpeB negative colonies

24 h	1/452
48 h	4/482
96 h	1/432

Figure S4. SpeB activity of animal-passaged *S. pyogenes*. (A) Detection of SpeB proteolytic activity by bacteria cultured in THY milk/agar. Black arrow indicates SpeB-negative colony. (B) Frequency of SpeB-negative colonies among animal-passaged *S. pyogenes*.

```
S.pyogenes_5448_WT        MENQKQKQKKYKNSLPKRLSNIFFVLFFCIFSAFTLIAYSSTNYFLLKKEKQSVFQAVNI
SpeB_negative_96_h_1      MENQKQKQKKYKNSLPKRLSNIFFVLFFCIFSAFTLIAYSSTNYFLLKKEKQSVFQAVNI
SpeB_negative_48_h_2      MENQKQKQKKYKNSLPKRLSNIFFVLFFCIFSAFTLIAYSSTNYFLLKKEKQSVFQAVNI
SpeB_negative_48_h_1      MENQKQKQKKYKNSLPKRLSNIFFVLFFCIFSAFTLIAYSSTNYFLLKKEKQSVFQAVNI

S.pyogenes_5448_WT        VRVRLSEVDSNFTLENLAEVLYKNDKTHLRIDDRKGSRVIRSERDITNTLDANQDIYVYN
SpeB_negative_96_h_1      VRVRLSEVDSNFTLENLAEVLYKNDKTHLRIDDRKGSRVIRSERDITNTLDANQDIYVYN
SpeB_negative_48_h_2      VRVRLSEVDSNFTLENLAEVLYKNDKTHLRIDDRKGSRVIRSERDITNTLDANQDIYVYN
SpeB_negative_48_h_1      VRVRLSEVDSNFTLENLAEVLYKNDKTHLRIDDRKGSRVIRSERDITNTLDANQDIYVYN

S.pyogenes_5448_WT        IDKQMIFTTDNEESSPGLHGPIGRVYHDHIEDQYRGFSMTQKVYSNRTGKFVGYVQVFHD
SpeB_negative_96_h_1      IDKQMIFTTDNEESSPGLHGPIGRVYHDHIEDQYRGFSMTQKVYSNRTGKFVGYVQVFHD
SpeB_negative_48_h_2      IDKQMIFTTDNEESSPGLHGPIGRVYHDHIEDQYRGFSMTQKVYSNRTGKFVGYVQVFHD
SpeB_negative_48_h_1      IDKQMIFTTDNEESSPGLHGPIGRVYHDHIEDQYRGFSMTQKVYSNRTGKFVGYVQVFHD

                                                                 Frame-shifts
S.pyogenes_5448_WT        LGNYYVIRARLLFWLLVVELFGTSLAYLIILITTRRFLKPLHNLHEVMRNISENPNNLNL
SpeB_negative_96_h_1      LGNYYVIRARLLFWLLVVELFGTSLAYLIILITTRRFLKPLHNLHEVMRNISENPNNLNL
SpeB_negative_48_h_2      LGNYYVIRARLLFWLLVVELFGTSLAYLIILITTRRF-----------------------
SpeB_negative_48_h_1      LGNYYVIRARLLFWLLVVELFGTSLAYLIILITTRRF-----------------------

                             D248Y
S.pyogenes_5448_WT        RSDISSGDEIEELSVIFDNMLDKLETHTKLQSRFISDVSHELRTPVAIIKGHIGLLQRWG
SpeB_negative_96_h_1      RSDISSGYEIEELSVIFDNMLDKLETHTKLQSRFISDVSHELRTPVAIIKGHIGLLQRWG
SpeB_negative_48_h_2      ------------------------------------------------------------
SpeB_negative_48_h_1      ------------------------------------------------------------

S.pyogenes_5448_WT        KDDSDILEESLTATAHEADRMAIMINDMLDMVRVQGSFEGHQNDMTVLEDSIETVVGNFR
SpeB_negative_96_h_1      KDDSDILEESLTATAHEADRMAIMINDMLDMVRVQGSFEGHQNDMTVLEDSIETVVGNFR
SpeB_negative_48_h_2      ------------------------------------------------------------
SpeB_negative_48_h_1      ------------------------------------------------------------

S.pyogenes_5448_WT        VLREDFIFTWQSENPKTIARIYKNHFEQALMILIDNAVKYSRKEKKIAINLSVTGKQEAI
SpeB_negative_96_h_1      VLREDFIFTWQSENPKTIARIYKNHFEQALMILIDNAVKYSRKEKKIAINLSVTGKQEAI
SpeB_negative_48_h_2      ------------------------------------------------------------
SpeB_negative_48_h_1      ------------------------------------------------------------

S.pyogenes_5448_WT        VRVQDKGEGISKEDIEHIFERFYRTDKSRNRTSTQAGLGIGLSILKQIVDGYHLQMKVES
SpeB_negative_96_h_1      VRVQDKGEGISKEDIEHIFERFYRTDKSRNRTSTQAGLGIGLSILKQIVDGYHLQMKVES
SpeB_negative_48_h_2      ------------------------------------------------------------
SpeB_negative_48_h_1      ------------------------------------------------------------

S.pyogenes_5448_WT        ELNEGSVFILHIPLAQSKES
SpeB_negative_96_h_1      ELNEGSVFILHIPLAQSKES
SpeB_negative_48_h_2      --------------------
SpeB_negative_48_h_1      --------------------
```

Figure S5. Amino acid sequence alignment of CovS of clones containing *covS* mutation. Only three clones among the animal-passaged *S. pyogenes* organisms showed a mutation in the CovS sequence. An amino acid substitution and frame-shifts are highlighted (yellow). Sequences were aligned using the ClustalW alignment program (https://www.genome.jp/tools-bin/clustalw).

4. Supplementary References

1. Maamary PG, Sanderson-Smith ML, Aziz RK, Hollands A, Cole JN, McKay FC, McArthur JD, Kirk JK, Cork AJ, Keefe RJ, Kansal RG, Sun H, Taylor WL, Chhatwal GS, Ginsburg D, Nizet V, Kotb M, Walker MJ. 2010. Parameters governing invasive disease propensity of non-M1 serotype group A streptococci. *J Innate Immun* 2:596-606.doi: 10.1159/000317640.
2. Mayfield JA, Liang Z, Agrahari G, Lee SW, Donahue DL, Ploplis VA, Castellino FJ. 2014. Mutations in the control of virulence sensor gene from *Streptococcus pyogenes* after infection in mice lead to clonal bacterial variants with altered gene regulatory activity and virulence. *PLoS One* 9:e100698.doi: 10.1371/journal.pone.0100698.
